# A Calibration Method for the Errors of Ring Laser Gyro in Rate-Biased Mode

**DOI:** 10.3390/s19214754

**Published:** 2019-11-01

**Authors:** Zengjun Liu, Lei Wang, Kui Li, Jingxuan Ban, Meng Wang

**Affiliations:** School of Instrumentation Science and Opto-electronics Engineering, Beihang University, Beijing 100191, China; lzj0601@buaa.edu.cn (Z.L.); eric.lee_buaa@buaa.edu.cn (K.L.); banjingxuan@126.com (J.B.); WangMeng_777@163.com (M.W.)

**Keywords:** gyroscopes, calibration, ring laser gyroscopes, angle random walk

## Abstract

Ring laser gyro (RLG) can work in mechanically dithered mode or rate-biased mode according to the working state of the inertial navigation system (INS). It can change from one mode to the other by receiving outer instructions. To evaluate the performance of RLG in rate-biased mode, an inertial measurement unit (IMU) based on RLG is installed on a dual-axis turntable, the turntable offers a constant angular velocity to the RLGs, in that way RLG can work in the rate-biased mode. A calibration method is proposed to calibrate the scale factor error, misalignments and constant bias of RLG in rate-biased mode, experiment results show that the differences of scale factor of the three gyros in two modes are 9 ppm, 7 ppm and 3.5 ppm, the constant biases of the three RLGs in rate-biased mode are also different from that in mechanically dithered mode with the difference of 0.017°/h, 0.011°/h and 0.020°/h, the input axis misalignment angle of RLGs in different modes also changed. What is more, a calculation method of angle random walk (ARW) of RLG in rate-biased mode is also presented. Experimental results show that the ARW of the RLG in rate-biased mode is about one third of that in mechanically dithered.

## 1. Introduction

**Figure 1:**
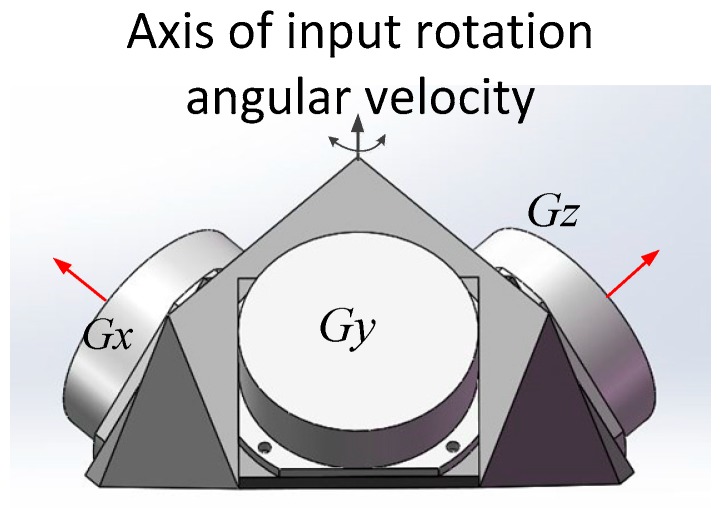
Structure of IMU based on RLG in rate-biased mode.

Ring laser gyroscope is a kind of inertial rotation sensor with the advantages of wide dynamic range, high reliability and high precision [1], which make RLG the ideal sensor for INS. Working on the principle of Sagnac effect, RLG has been used widely in many navigation and guidance fields [2,3,4]. Different from fiber optic gyroscopes, RLG has an obvious lock-in phenomenon [5], where beat signal disappears at low rotation rates due to back-scattering lights inside the ring cavity. Many techniques have been developed to overcome the lock-in problem [6], and the most widely used is the mechanically dithering technology. However, dither cannot truly solve the lock-in problem, as each time the dither direction reverses, a short interval exists when the rotation rate is near zero and lock-in problem will occur. In fact, ARW is mainly caused by this lock-in phenomenon occurring in the mechanically dithered RLG. The rate-biased technique utilizes an extra turntable to offer a constant angular velocity to the RLG, where the angular velocity of the gyro is always beyond the lock-in threshold. By this way, the lock-in problem is fixed completely, and ARW of RLG is also reduced [7]. However, adding a turntable increases the complexity of the INS on one hand; on the other hand, the angular velocity of the turntable cannot approximate the angular velocity of the carrier of INS; if they rotate in different direction with the same magnitude, the lock-in problem will reoccur. Due to the above two aspects, the applications of the rate-biased RLG are limited. In spite of the limits, many single-axis high precision rotation inertial navigation systems (RINS) still use rate-biased RLG. RLGs are usually installed obliquely so that a single turntable can meet the need of three rate-biased RLGs. As shown in Figure 1, the angles between the gyro and the axis of input rotation angular velocity are the same, which are around 54.74° [8].

To combine the advantages of the two techniques, mechanically dithered/rate-biased RLG was developed. It can work in mechanically dithered mode or rate-biased mode according to the application environment. The different working mode will cause different characteristics and there are mature methods to calibrate the errors of RLG in the mechanically dithered mode [9,10], however, there are few calibration methods to evaluate the performance of RLG in rate-biased mode, because the turntable must rotate with a high angular velocity to make the RLGs stay in rate-biased mode, in this paper, a calibration method is proposed to calibrate the errors of RLGs in rate-biased mode, what is more, a ARW calculation method of RLG in rate-biased mode is also proposed. The RLGs tested in the paper are custom devices.

For the mechanically dithered RLG, the most widely used method to calculate the ARW of a single RLG was reported in the IEEE Std 647TM-2006 [11,12]. This method is based on Allan variance, which can be applied to analyze the error characteristics of any precision measurement instruments. Besides this, another method based on classic variance uses a three-order polynomial to fit the errors to calculate the ARW coefficient, which was proposed by the Honeywell [13]. However, the second method is only an empirical formula, which does not explain the sources of the errors. For the RLG in rate-biased mode, ARW is also difficult to calculate just like the case of bias, where the errors caused by the rotation are hard to be eliminated. A method based on fast orthogonal search (FOS) was reported in reference [8], which calculates the average ARW coefficient of three constant rate-biased RLG in RINS system. The structure of the three gyros is shown in Figure 1. This method assumes that the three gyro outputs all contain the errors caused by rotation, and the difference of every two gyros outputs will deduct these errors. However, there are problems in this method. FOS method will remove all the periodic components including the components of high frequency which may be caused by the electronic circuit of the RLG itself, while these components of high frequency will affect the performance of RLG in the system and we should not remove them when RLG is evaluated. ARW coefficient is the standard deviation of the white noise in the angular velocity signal output of the RLG, if we calculate the ARW of the sum or the difference of two gyros, the relationship of them should be described as Equation (1).

(1) $$ARW_{sum/difference}=\sqrt{ARW_{g1}{}^{2}+ARW_{g2}{}^{2}}\neq \frac{1}{2}(ARW_{g1}+ARW_{g2})$$

Here, $ARW_{g1}$ stands for the ARW of the first gyro, $ARW_{g2}$ represents the ARW of the second gyro, $ARW_{sum/difference}$ means the ARW of the sum or the difference of the two gyros outputs. From Equation (1), it is clear that the difference of two gyro outputs cannot be used to calculate the average ARW coefficient, so the method proposed in reference [8] has a few drawbacks. What is more, this method cannot be used in evaluating the performance of a single RLG in rate-biased mode. 

Fourier analysis is an effective method to analyze the periodic components in a signal. In this paper, this method is also adopted. When the data series of gyro is acquired, the main frequencies of the periodic rotation error in every revolution are obtained by applying Fourier analysis. Then least square fitting is made to obtain the function of these periodic errors versus rotation angle. The fitted results are subtracted from the original gyro outputs. Apart from that, during the experiment, some errors, such as the misalignments and the scale factor error, will change slowly with the increasing temperature. And these errors should also be removed before the calculation of ARW. 

The remaining of this paper are organized as follows: Section 2 describes the characteristics of the IMU used and the RLG characteristics in mechanically dithered mode as well as experimental equipment, while discussions about the scale factor will be presented in Section 3. Section 4 describes the calibration methods of IMU; methods to calculate the ARW coefficients of RLG in rate-biased mode will be presented in Section 5. Conclusions will be drawn in Section 6.

## 2. Characteristics of the IMU

The specifications of RLG in mechanically dithered mode are shown in Table 1.

**Figure 2:**
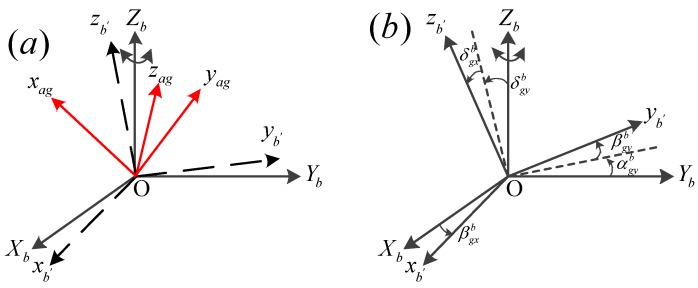
Frames of the IMU. (**a**) The relationship between a frame, b frame and $b^{\prime }$ frame; (**b**) The errors angles between b frame and $b^{\prime }$ frame.

The frames of the IMU are shown in Figure 2a. The a frame is defined by the gyros’ input axis, b-frame is defined as follows, $Z_{b}$ coincides with the ideal axis of input rotation angular velocity, the $X_{b}$ lies along the projection of gyro sensitive axis $x_{a}$ on the normal plane of $Z_{b}$, and $Y_{b}$ is defined according to the right-hand rule. All the errors including the misalignments, scale factor errors and biases of the inertial devices are all defined in b frame for simplicity, so anther $b^{\prime }$ frame is defined between the a-frame and the b-frame, and it likes that the RLGs and the accelerometers are installed in $b^{\prime }$ frame.

The outputs in b-frame can be described as:

(2) $$\left[\begin{array}{c} \omega_{gx}^{b}\\ \omega_{gy}^{b}\\ \omega_{gz}^{b} \end{array}\right]=C_{b^{\prime }}^{b}C_{ag}^{b^{\prime }}\left[\begin{array}{c} N_{gx}\\ N_{gy}\\ N_{gz} \end{array}\right]$$

where, $N_{gx}$, $N_{gy}$ and $N_{gz}$ are the original outputs of the RLG.

(3) $$C_{ag}^{b^{\prime }}=\left[\begin{array}{ccc} \frac{\sqrt{6}}{3} & -\frac{\sqrt{6}}{6} & -\frac{\sqrt{6}}{6}\\ 0 & -\frac{\sqrt{2}}{2} & \frac{\sqrt{2}}{2}\\ -\frac{\sqrt{3}}{3} & -\frac{\sqrt{3}}{3} & -\frac{\sqrt{3}}{3} \end{array}\right]$$

The direction cosine matrix from $b^{\prime }$ frame to b frame is described as:

(4) $$C_{b^{\prime }}^{b}=\;\left[\begin{array}{ccc} 1 & 0 & \beta_{gx}^{b}\\ \alpha_{gy}^{b} & 1 & -\beta_{gy}^{b}\\ -\delta_{gy}^{b} & \delta_{gx}^{b} & 1 \end{array}\right]\left[\begin{array}{c} k_{gx}^{b}\left(1+\Delta k_{gx}^{b}\right)\\ k_{gy}^{b}\left(1+\Delta k_{gy}^{b}\right)\\ k_{gz}^{b}\left(1+\Delta k_{gz}^{b}\right) \end{array}\right]$$

The five small angles $\beta_{gx}^{b}$, $\beta_{gy}^{b}$, $\alpha_{gy}^{b}$, $\delta_{gx}^{b}$, $\delta_{gy}^{b}$ represent the misalignments of gyros and the five angles are shown in Figure 2b, $k_{gx}^{b}$, $k_{gy}^{b}$, $k_{gz}^{b}$ represent the scale factor of the gyros in b-frame and $\Delta k_{gx}^{b}$, $\Delta k_{gy}^{b}$ and $\Delta k_{gz}^{b}$ represent the scale factor errors. The errors of the accelerometers include six misalignments, three scale factor errors and three constant biases, they are similar with the errors of gyros, so they are not described here in detail. In this way, the errors are defined in the same way with the non-obliquely installed IMU. In this paper, we focus on the characteristics of the RLG in rate-biased mode, so the errors of RLG in mechanically dithered mode and the errors of the accelerometer including the lever arm effect should be calibrated in advance [9,10,14]. After the calibration, the scale factor errors of accelerometers will be less than 2 ppm, the constant biases of accelerometers will be less than 5 μg the misalignments of accelerometers will be less than 2″, the residual errors of accelerometers will have little effects on the calibration of the errors of RLG in rate-biased mode.

**Figure 3:**
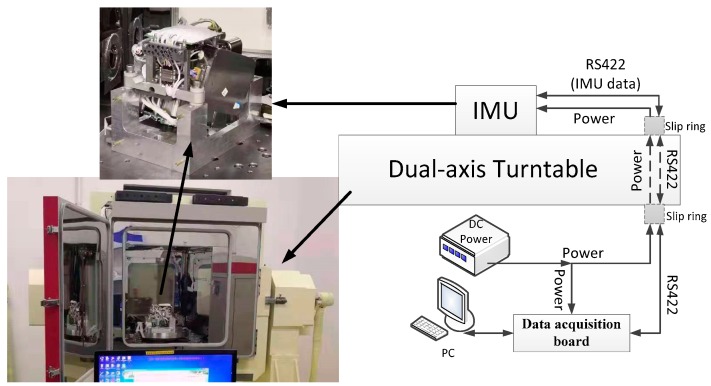
Experimental setup.

As described in the former parts of this paper, the RLG has two modes including rate-biased mode and mechanically dithered mode, which can be controlled by the data acquisition board of IMU. When the IMU begins to work in the static state, RLGs will be in the mechanically dithered mode first, when IMU is fixed on the turntable, which will rotate along inner axis and after the angular velocity of the rotation is beyond the lock-in threshold, the board will send instructions to RLGs control board and RLGs will switch to the rate-biased mode. The experimental setup is shown in the Figure 3, the IMU is rigidly fixed in the turntable and the IMU outputs are collected by a computer, it is important to note that the rotation angles of the turntable are collected at the same time. There is a synchronic signal between the turntable and the data acquisition board. The frequency of the data collection is 200 Hz. The turntable has slip rings can transmit the data from IMU to the data acquisition board, and serial communication protocol (Rs-422) is used during the transmission. The information of the turntable is shown in Table 2 [15].

## 3. Scale Factor Error

The calibrated errors of RLG in rate-biased errors including the scale factor errors, the bias of RLG and the misalignments of RLG in rate-biased mode are compared to these errors in mechanically dithered mode which are calibrated well in advance. The scale factor of RLG in rate-biased mode has a close relationship with input angular velocity [1] and it is easy to calibrate the scale factor error $\Delta k_{gz}^{b}$ in rate-biased mode using the method which uses the turntable to provide a known angular velocity to the RLG, then the scale factor of RLG can be calibrated according to the output of RLG and the known angular velocity, we calibrate the $\Delta k_{gz}^{b}$ with the angular velocity of $1^{\circ }/s$ $2^{\circ }/s$, $3^{\circ }/s$, $6^{\circ }/s$, $9^{\circ }/s$, $12^{\circ }/s$, $15^{\circ }/s$, $24^{\circ }/s$, $30^{\circ }/s$, $40^{\circ }/s$, $50^{\circ }/s$, and $60^{\circ }/s$. The experiment has been conducted for 5 times to minimize the chance error; results are shown in Table 3.

**Figure 4:**
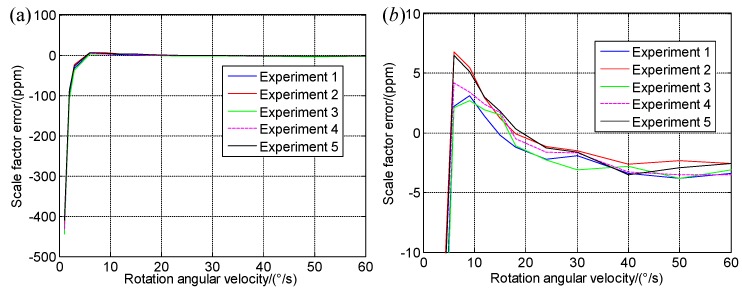
$\Delta k_{gz}^{b}$ of the IMU in rate-biased mode; (**a**) $\Delta k_{gz}^{b}$ with the change of the rotation angular velocity; (**b**) Partial enlargement of (a).

The results are plotted in a line chart to demonstrate the changes more clear, as shown in Figure 4.

From Table 3 and Figure 4, as the rotation angular velocity increases, the scale factor becomes more stable, when the rotation velocity is more than $30^{\circ }/s$, the difference of the scale factors of RLG in two modes is around 3 ppm, when the rotation angular velocity is beyond $12^{\circ }/s$, the standard deviation of the scale factor errors with a fixed angular velocity is less than 1ppm, so that the input angular velocity of $Z_{b}$ should be more than $15^{\circ }/s$, and $30^{\circ }/s$ will be chosen in this paper.

## 4. Calibration Method of RLG in Rate-Biased Mode

**Figure 5:**
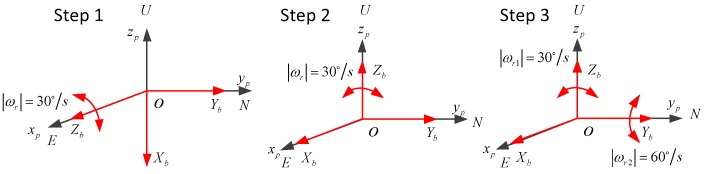
Three steps of the calibration.

To calibrate the errors of RLG in rate-biased mode, a dual-axis or tri-axis turntable is needed. The initial position frame of dual-axis turntable denoted as p is defined by the directions of the two-rotation axis of the dual-axis turntable. When IMU is installed on the turntable, the inner axis of turntable coincides with $Z_{b}$ approximately; the outer axis of turntable coincides with $Y_{b}$ approximately. Navigation frame representing local-level geographic coordinate frame is denoted as n. As shown in Figure 1, the three gyros are always installed obliquely; the dual-axis turntable must always rotate along the inner axis to offer angular velocity to the three RLGs during the calibration. To reduce the coupling of the different errors, the calibration method proposed here will be divided into three steps shown in Figure 5.

The first step is that the outer axis of the turntable locks at $90^{\circ }$, the inner axis rotates for ten revolutions and then rotate in the reverse direction for another ten revolutions, the turntable repeats the rotation for 5 times, the angular velocity of the rotation is $30^{\circ }/s$.

During this step, the angular velocity errors in n-frame denoted as $\Delta \omega^{n}$ can be described as:

(5) $$\Delta \omega^{n}=\left[\begin{array}{c} \Delta \omega_{E}^{n}\\ \Delta \omega_{N}^{n}\\ \Delta \omega_{U}^{n} \end{array}\right]\approx \left[\begin{array}{c} \beta_{gx}^{b}\omega_{r}\cos\omega_{r}t+\beta_{gy}^{b}\omega_{r}\sin\omega_{r}t\\ \Delta k_{gz}^{b}\omega_{r}+\varepsilon_{gz}^{b}\\ \beta_{gx}^{b}\omega_{r}\sin\omega_{r}t-\beta_{gy}^{b}\omega_{r}\cos\omega_{r}t \end{array}\right]$$

where, $\omega_{r}$ is the rotation angular velocity and t is the time. The angle errors in horizontal direction denoted as $\Delta \phi_{E}$, $\Delta \phi_{N}$ can be obtained by integrating $\Delta \omega_{E}^{n}$ and $\Delta \omega_{N}^{n}$.

(6) $$\{\begin{array}{l} \Delta \phi_{E}=\int_{0}^{T}\Delta \omega_{E}^{n}dt=\beta_{gx}^{b}\sin\omega_{r}T+\beta_{gy}^{b}(1-\cos\omega_{r}T)\\ \Delta \phi_{N}=\int_{0}^{T}\Delta \omega_{N}^{n}dt=\Delta k_{gz}^{b}\omega_{r}T+\varepsilon_{gz}^{b}T \end{array}$$

The velocity errors can be described as:

(7) $$\{\begin{array}{l} \Delta V_{E}=\frac{-g(1-\cos\omega_{r}T)}{\omega_{r}}\beta_{gx}^{b}-g\beta_{gy}^{b}(\frac{\omega_{r}T+\sin\omega_{r}T}{\omega_{r}})\\ \Delta V_{N}=\frac{1}{2}g\Delta k_{gz}^{b}\omega_{r}T^{2}+\frac{1}{2}g\varepsilon_{gz}^{b}T^{2} \end{array}$$

where, g is the acceleration of gravity and T is the calibration time. Based on the analyses above, recursive least squares (RLS) could be employed for the optimal estimation of $\Delta k_{gz}^{b}$, $\varepsilon_{gz}^{b}$, $\beta_{gx}^{b}$ and $\beta_{gy}^{b}$ the state model could be defined as

(8) $$X={\left[\begin{array}{cccc} \Delta k_{gz}^{b} & \varepsilon_{gz}^{b} & \beta_{gx}^{b} & \beta_{gy}^{b} \end{array}\right]}^{T}$$

The discretized measurement equation could be written as:

(9) Z=HX+V

where,

(10) $$Z=\left[\begin{array}{c} \Delta V_{E}\\ \Delta V_{N} \end{array}\right]$$

(11) $$H=\left[\begin{array}{cc} \frac{1}{2}g\omega_{r}T & \frac{1}{2}gT^{2}\\ \frac{-g(1-\cos\omega_{r}T)}{\omega_{r}} & -g(\frac{\varphi +\sin\omega_{r}T}{\omega_{r}}) \end{array}\right]$$

RLS algorithm could be employed as shown in Equation (12).

(12) $$\begin{array}{l} P_{k+1}=P_{k}-P_{k}H_{k+1}^{T}{\left(I+H_{k+1}P_{k}H_{k+1}^{T}\right)}^{-1}H_{k+1}P_{k}\\ X_{k+1}=X+kP_{k+1}H_{k+1}^{T}(Z_{k+1}-H_{k+1}X_{k}) \end{array}$$

In the step 2, $\varepsilon_{gx}^{b}$ and $\varepsilon_{gy}^{b}$ are calibrated, which are the constant biases of RLGs in b-frame. A calibration method is proposed in reference [16], however, it cannot be used here, for the turntable must rotate along the inner axis to keep the RLGs in the rate-biased mode. The rotation scheme of this step is that the turntable rotates along the inner axis for ten revolutions, then rotates for another ten revolutions in the reverse direction, after that repeat this process for 5 times. During this step the angular velocity in p frame can be described as:

(13) $$\left\{ \begin{array}{l} \omega_{px}=\varepsilon_{gx}^{b}\cos\phi -\varepsilon_{gy}^{b}\sin\phi +\omega_{px0}\\ \omega_{py}=\varepsilon_{gx}^{b}\sin\phi +\varepsilon_{gy}^{b}\cos\phi +\omega_{py0} \end{array}\right.$$

where, ϕ is the rotation angle of the inner gimbal of the turntable, in this way, recursive least squares (RLS) could be employed for the optimal estimation of $\varepsilon_{gx}^{b}$ and $\varepsilon_{gy}^{b}$.

During the step 3, the inner gimbal and the outer gimbal of the turntable rotate at the same time, the angular velocity of the inner axis is $30^{\circ }/s$, the angular velocity of the outer axis is $60^{\circ }/s$, just as in step 1, the relationship between the velocity errors and the errors can be described as:

(14) $$\{\begin{array}{l} \begin{array}{ll} \Delta V_{E} & =-g\delta_{gx}^{b}(\frac{\theta +\sin\theta }{\omega_{r}})\cos\phi \\ & +(\frac{-g(1-\cos\theta )}{\omega_{r}}\alpha_{gy}^{b}-g\delta_{gy}^{b}(\frac{\theta +\sin\theta }{\omega_{r}}))\sin\phi \end{array}\\ \begin{array}{cc} \Delta V_{N} & =(\frac{1}{2}g\Delta k_{gy}^{b}\omega_{r}T^{2}+\frac{1}{2}g\varepsilon_{gy}^{b}T^{2})\cos\phi \\ & +(\frac{1}{2}g\Delta k_{gx}^{b}\omega_{r}T^{2}+\frac{1}{2}g\varepsilon_{gx}^{b}T^{2})\sin\phi \end{array} \end{array}$$

where, $\Delta V_{E}$ and $\Delta V_{N}$ is the velocity errors in the east and north direction, $\omega_{r}$ is the rotation angular velocity of the outer gimbal $\left|\omega_{r}\right|=60^{\circ }/s$, T is the calibration time of step 3, θ is the rotation angle of the outer gimbal, based on the Equation above, as $\varepsilon_{gx}^{b}$ and $\varepsilon_{gy}^{b}$ have been calibrated, it is known that the $\Delta k_{gx}^{b}$, $\Delta k_{gy}^{b}$, $\alpha_{gy}^{b}$, $\delta_{gx}^{b}$ and $\delta_{gy}^{b}$ can be calibrated using RLS method. 

The calibration results are shown in Table 4. 

**Figure 6:**
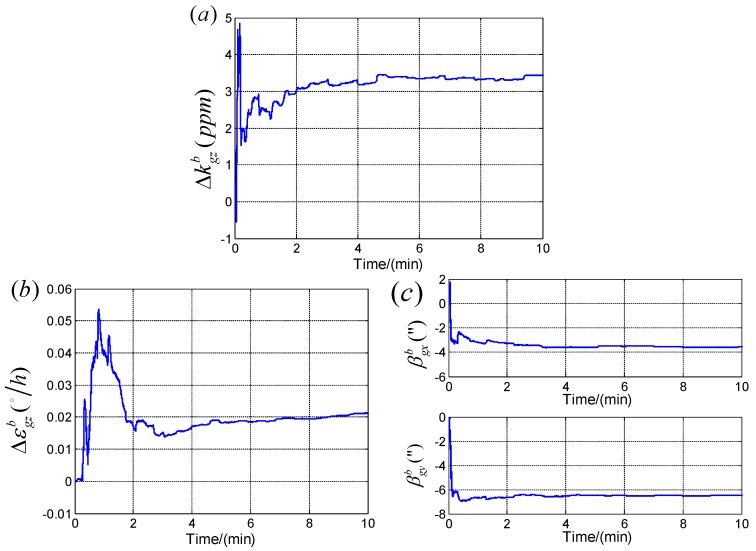
The curve convergences of the calibrated errors in the first step. (**a**) The curve convergences of $\Delta k_{gz}^{b}$; (**b**) The curve convergences of $\varepsilon_{gz}^{b}$; (**c**) The curve convergences of $\beta_{gx}^{b}$ and $\beta_{gy}^{b}$.

The curve convergences of the calibrated errors in first step are taken as examples shown in Figure 6.

From the calibration results, the differences of scale factor errors in b-frame of RLGs in two modes are 9 ppm, 7 ppm and 3.5 ppm, the differences of the biases of RLGs in b-frame are about $0.017^{\circ }/h$, $0.011^{\circ }/h$ and $0.02^{\circ }/h$, the misalignments in b frame are also different, which may be caused by the changing of the input axis misalignment angle of RLGs in different modes [17].

**Figure 7:**
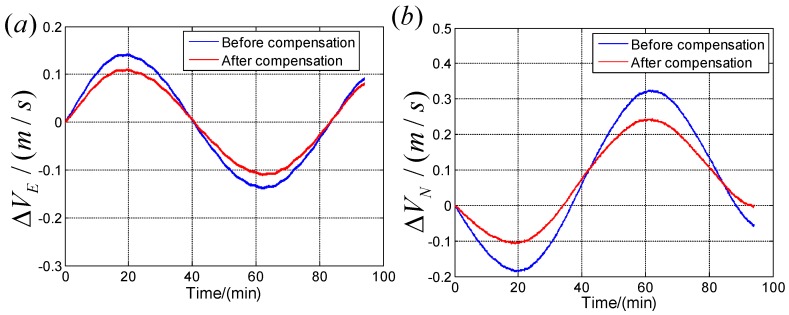
Navigation results before and after compensating the calibration results. (**a**)Velocity errors in east direction compensation; (**b**) Velocity errors in north direction.

To further verify the calibration results, navigation experiments are conducted, the rotation scheme is the same with the step 3. The velocity errors are shown in Figure 7, and it is known that the calibration result compensations are effective.

## 5. Calculation of Angle Random Walk

**Figure 8:**
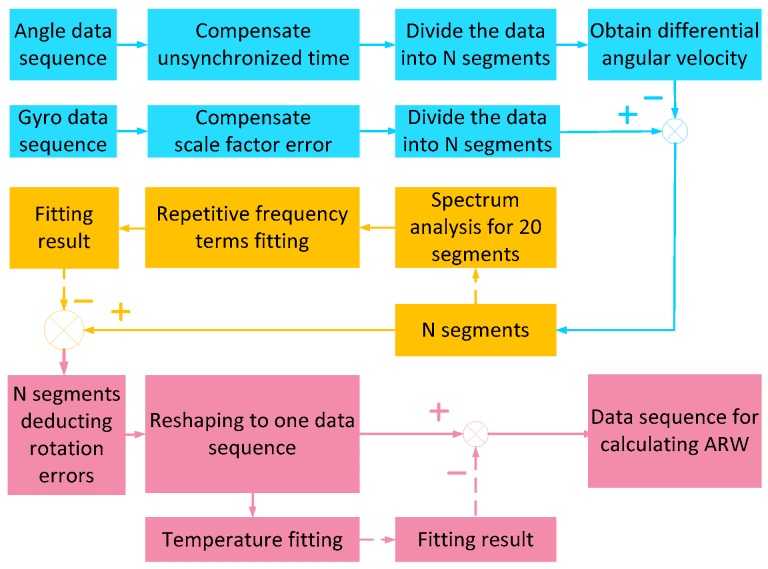
Flow of the process.

To calculate the ARW of RLG in rate-biased mode, the turntable rotates along the inner axis clockwise for about 5 h, the gyro outputs and the rotation angle are collected at the same time. Two groups of experiment with angular velocity of $30^{\circ }/s$ and $60^{\circ }/s$ are conducted respectively. When the data series is acquired, the data should be pre-processed before calculating the ARW of the RLGs. The aim of the pre-processing is to remove the periodic errors caused by the rotation. The experiment with angular velocity of $60^{\circ }/s$ is taken as an example to show the pre-processing. The data processing flow of $\omega_{gz}^{b}$ is shown in Figure 8 below.

**Figure 9:**
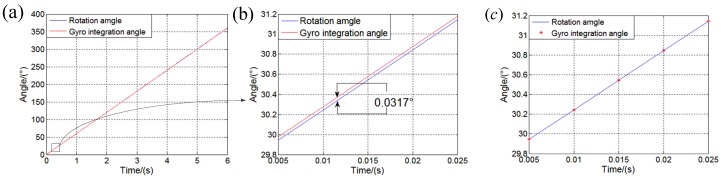
Unsynchronized time compensation results. (**a**) Before compensation; (**b**) Partial enlargement of (a); (**c**) Results after compensation.

During the experiment, a synchronic signal is used to make sure the rotation angle and the IMU outputs are kept synchronous. However, they cannot be collected exactly at the same time. Hence, it is necessary to make compensations. When the asynchronous time is calculated, it is assumed that the rotation angular velocity in short time is constant. If there is no delay between them, the rotation angle of the turntable and the integration of $\omega_{gz}^{b}$ will coincide. However, as shown in Figure 9a,b, the difference between the rotation angle and the integration angle of $\omega_{gz}^{b}$ is about $0.0317^{\circ }$ in every 5 ms, which means a delay exists in the rotation angle. The delay Δt can be calculated as:

(15) $$\Delta t=0.0317^{\circ }/(60^{\circ }/s\times 0.005\;s)\times 5\;ms=0.5283\mathrm{ms}$$

If we use R(k) to stand for the rotation angle in the time k, the compensation for the delay can be described as:

(16) R(k)=R(k)×(1−Δt/5)+R(k+1)×(Δt/5)

The compensation result is shown in the Figure 9c, and the rotation angle and the gyro integration angle coincide well.

**Figure 10:**
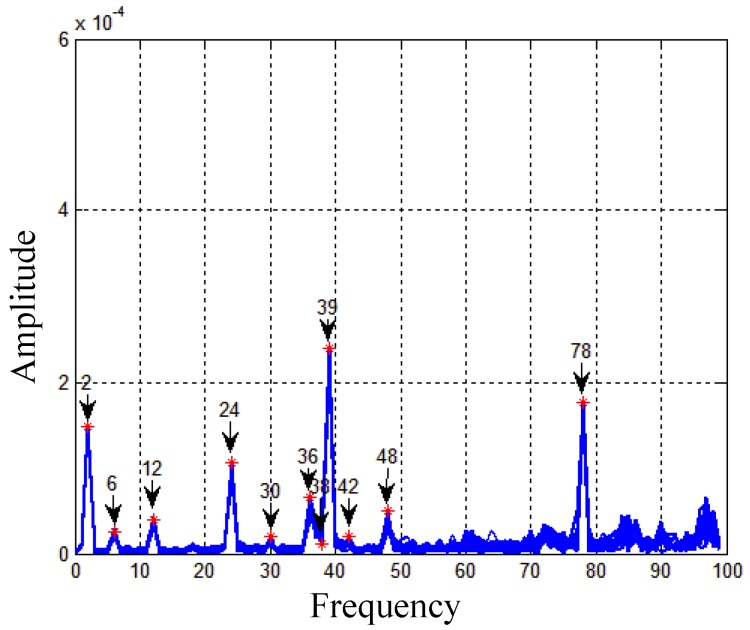
Spectrum analysis.

In the one-time experiment, the turntable rotates for about 3000 revolutions. In every revolution, the characteristics of the rotation errors caused by the rotation are similar. Therefore, the data sequence is possible to be reshaped into 3000 short data segments to remove periodic errors. Fourier analysis is used to analyze the main frequencies of the periodic errors [18]. The frequency spectrum is shown in Figure 10, where 20 short segments are analyzed to guarantee that the fitting model is correct.

Form Figure 10, it is known that the main frequencies are $2\omega_{0}$, $6\omega_{0}$, $12\omega_{0}$, $24\omega_{0}$, $30\omega_{0}$, $36\omega_{0}$, $38\omega_{0}$ $39\omega_{0}$, $48\omega_{0}$, and $78\omega_{0}$. Here, $\omega_{0}$ stands for the fundamental frequency. There are some other frequencies which are not repetitive in the 20 revolutions. But they are not taken into consideration for these errors contribute little to the calculation of ARW. Then the rotation errors in every revolution can be fitted using the equation based on Least squares fitting [19].

(17) $$\begin{array}{cc} fft\_result & =a_{1}\cos\left(2R_{k}\right)+b_{1}\sin\left(2R_{k}\right)+\ldots \\ & \ldots +a_{10}\cos\left(78R_{k}\right)+b_{10}\sin\left(78R_{k}\right) \end{array}$$

In the equation above, $R_{k}$ stands for the rotation angle of the kth revolution, $a_{1},b_{1},\ldots a_{10},b_{10}$ represent the coefficients for fitting. When these coefficients are obtained, the rotation errors will be subtracted from every gyro segment. After that, these short segments could be reshaped into the long data sequence again.

The last step is to deduct the errors that vary with temperature, because during the experiment, temperature will be increasing all the time, and some errors will be changed with that. The data series is averaged with a period of 6 seconds for the temperature changes slowly, and then a five-order polynomial will be used to fit the trend term.

**Figure 11:**
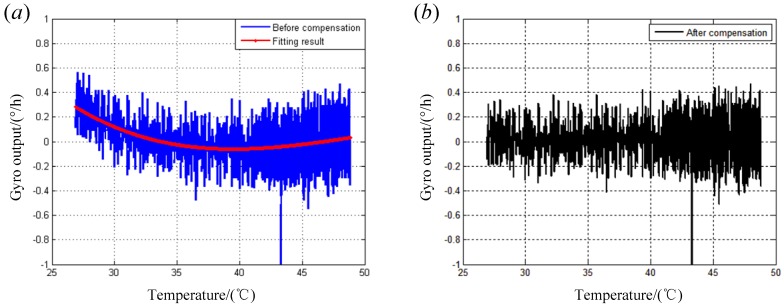
Temperature compensation result; (**a**) The fitting result of the data; (**b**)The result after temperature compensation.

When the polynomial is obtained, it is to be subtracted from the long data series. The compensation result is shown in Figure 11 below.

**Figure 12:**
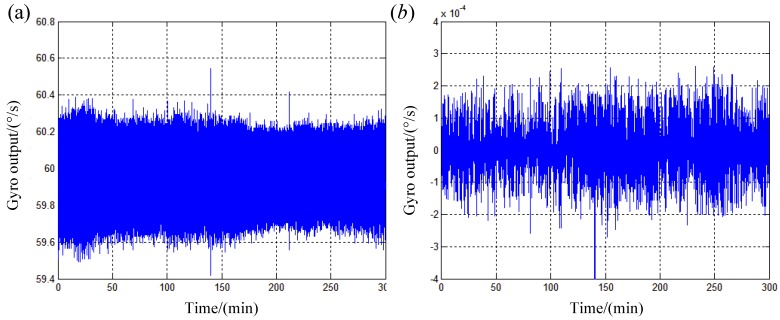
Gyro outputs for calculating ARW. (**a**) Original gyro outputs; (**b**) Gyro outputs after pre-processing.

Finally, the long data series is averaged with a period of one second which will be used to calculate the ARW shown in Figure 12b. Comparing original gyro outputs and gyro outputs after pre-processing shown in Figure 12, we can see that the method proposed in this study is effective.

The method used to calculate the ARW was described in reference [12] in detail. In brief, the Allan variances for different averaging time τ of the data series which was obtained in last step are firstly calculated, and then the relationship between the different variances and the corresponding averaging time is fitted by a five-order polynomial described in Equation (18), ARW coefficient is $\sqrt{N}$. How to calculate the Allan variance for averaging time τ will not be described here.

(18) $$\sigma_{Allan}{}^{2}(\tau )=\frac{R^{2}\tau^{2}}{2}+\frac{K^{2}\tau }{3}+\frac{2\ln2}{\pi }B^{2}+\frac{N^{2}}{\tau }+\frac{3Q^{2}}{\tau^{2}}$$

**Figure 13:**
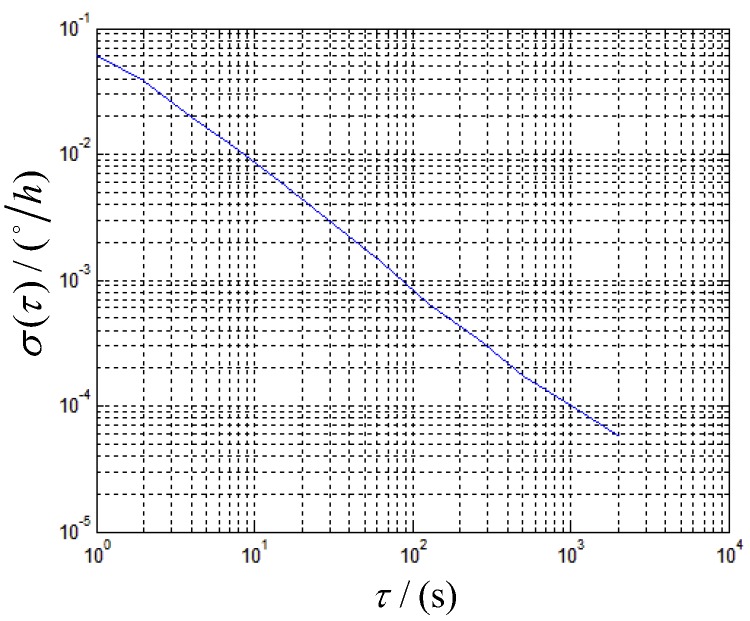
Root Allan variance versus averaging time for gyro in rate-biased mode.

The $\sigma_{Allan}{}^{2}(\tau )$ versus τ is shown in Figure 13.

The ARW of $\omega_{gz}^{b}$ in rate-biased mode with the angular velocity of $60^{\circ }/s$ is about $0.0004^{\circ }/\sqrt{h}$. The other groups of experiment with the rotation angular velocity of $30^{\circ }/s$ are also processed by this method, the results are listed in Table 5.

To further verify this method, the same experiments for RLG in mechanically dithered mode are also conducted. The ARW are also calculated with the same method, and the results are listed in Table 6.

From Table 6, it is clear that no matter the turntable rotates or not, the ARW of the gyro in mechanically dithered mode is about $0.0013^{\circ }/\sqrt{h}$, and the value is similar with the given value listed in Table 1, this proves the method used in this paper is valid. From Table 5, it can be seen that the ARW of the gyro in rate-biased mode is about $0.0004^{\circ }/\sqrt{h}$, which does not change with the rotation angular velocity.

To further verify the calculation method, alignment experiments are also conducted. When the alignment experiments are conducted, the inner gimbal of the turntable rotates for 20 revolutions then rotates in the reverse direction for another 20 revolutions, the angular velocity is set to be $60^{\circ }/s$, the inner gimbal repeats the rotation strategy for 40 min. The middle and outer gimbal lock at initial position. The alignment experiments are conducted for 2 groups when the RLGs are in rate-biased mode and mechanically dithered mode, each group includes 15 times. There are many mature fine alignment models that can be used [20,21], which is not described here. Offline program was used to process the alignment experiments data; standard deviation of azimuth angle in n-frame for 12 min, 16 min, 20 min, 24 min, 28 min, 32 min, 36 min and 40 min can be obtained.

Based on the rotation strategy above, ARW of the two RLGs in horizontal direction is one of the key factors to the fine alignment accuracy, the standard deviation of azimuth angle in n frame at one fixed position of 15 times experiments can be calculated as:

(19) $$STD_{Azimuth}=(\frac{{\bar{ARW}}^{b}}{\sqrt{T}}+{\tilde{\varepsilon }}_{E}^{b})/(\omega_{ie}\cos(L))$$

where, T is the time of alignment, ${\bar{ARW}}^{b}$ is the mean value of the ARW of the two RLGs in horizontal direction in b frame, ${\tilde{\varepsilon }}_{E}^{b}$ is sum of other factors which will affect the alignment accuracy, $\omega_{ie}$ is the rotational angular velocity of the earth, L is the latitude. Experiment results are listed in Table 7.

**Figure 14:**
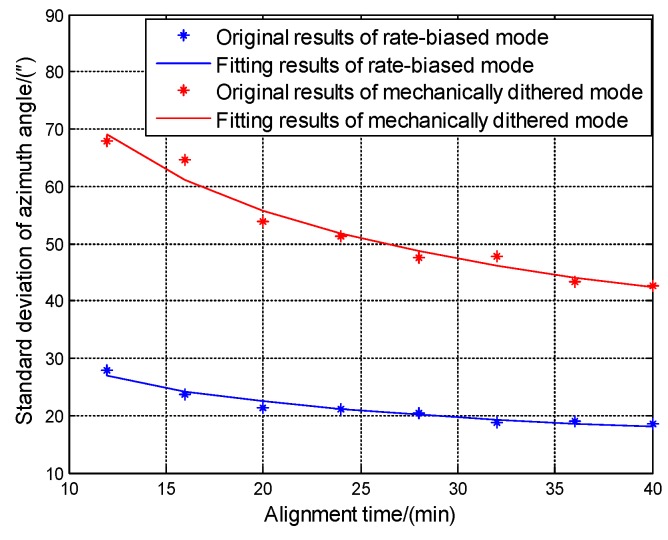
Fitting results of standard deviation of azimuth angle verse T in alignment experiments.

From Table 7, it is known that as the time of alignment becomes longer, the standard deviation of azimuth angle in n frame decreases, because the effect of $\frac{{\bar{ARW}}^{b}}{\sqrt{T}}$ decreases as T increases. The experiment results in Table 7 can be used to fit Equation (19) to obtain the ${\bar{ARW}}^{b}$ in different mode, ${\bar{ARW}}^{b}$ of rate-biased mode is $0.00041^{\circ }/\sqrt{h}$ and ${\bar{ARW}}^{b}$ of mechanically dithered mode is $0.00134^{\circ }/\sqrt{h}$, the fitting results are shown in Figure 14 below.

From the Table 5, it is known that the ARW calculation results of RLGs in rate-biased match with the azimuth angle accuracy of the alignment experiments, which further prove the calculation method is effective.

## 6. Conclusions

In this paper, methods about how to evaluate the RLG in rate-biased mode are proposed, and the main characteristics of the RLG in the two modes are compared. The scale factor of the gyro is discussed firstly, and experiments are conducted to calibrate different rotation angular velocities’ scale factor error of the gyro in rate-biased mode. Based on the experiment results, as the rotation angular velocity increases, the scale factor becomes more stable, when the rotation velocity is more than $30^{\circ }/s$, the difference of the scale factors of RLG in two modes is around 3 ppm, and $30^{\circ }/s$ is chosen in this paper to calibrate the errors and the calculate the ARW of the RLG in rate-biased mode. Afterwards, a calibration method for errors of RLG including constant bias, scale factor error and misalignments in rate-biased mode is proposed, experiment results show that the differences of these errors of RLG in two modes exist obviously, especially for the constant bias. At last, a method based on Fourier analysis calculating ARW of the gyro in rate-biased mode is proposed. Using the calculation method the ARW of RLG in mechanically dithered mode is about $0.0013^{\circ }/\sqrt{h}$ while in the rate-biased mode, the result is about $0.0004^{\circ }/\sqrt{h}$, which is about one third of that in mechanically dithered mode.

## Figures and Tables

**Table 1:** Specifications of RLG.

Characteristics (Mechanically Dithered Mode)	Values
Output frequency	200 Hz
Gyro bias stability	$0.007^{\circ }/h$
ARW	$0.0012^{\circ }/\sqrt{h}$
Gyro scale factor	≈0.011 Hz/(rad/s)
Gyro scale factor stability	3 ppm

**Table 2:** Specifications of dual-axis turntable.

Characteristics	Values
Position angle resolution	$<3^{\prime \prime }$
Angular velocity stability	$<0.001{}^{\circ }/s$
Maximal angular speed	$\pm 400^{\circ }/s$
Maximal angular acceleration	$100^{\circ }/s^{2}$
Non-orthogonality of the rotation axes	$<3^{\prime \prime }$
Maximum weight load	50 kg

**Table 3:** Calibration results of $\Delta k_{gz}^{b}$ in rate-biased mode using traditional method.

Rotation Angular Velocity $\left({}^{\circ }/s\right)$	$\Delta k_{gz}^{b}$ (ppm)					Standard Deviation (ppm)
	1	2	3	4	5	
1	−431.4	−421.9	−445.1	−432.4	−409.8	13.2
2	−98.4	−87.9	−110.2	−86.4	−87.5	10.2
3	−32.1	−23.6	−37.2	−25.4	−27.9	5.5
6	2.2	6.8	2.1	4.2	6.5	2.3
9	3.1	5.5	2.7	3.4	5.1	1.3
12	1.4	2.9	1.9	2.4	3	0.7
15	−0.2	1.2	1.5	1.7	1.8	0.8
18	−1.2	−0.1	−1.1	−0.5	0.3	0.6
24	−2.2	−1.14	−2.3	−1.6	−1.3	0.5
30	−1.9	−1.52	−3.1	−1.7	−1.6	0.6
40	−3.4	−2.62	−2.8	−3.3	−3.5	0.4
50	−3.8	−2.35	−3.8	−3.5	−2.9	0.6
60	−3.4	−2.56	−3.1	−3.5	−2.6	0.4

**Table 4:** Calibration results.

Angular Velocity	$\begin{array}{l} \Delta k_{gx}^{b}\\ \left(ppm\right) \end{array}$	$\begin{array}{l} \Delta k_{gy}^{b}\\ \left(ppm\right) \end{array}$	$\begin{array}{l} \Delta k_{gz}^{b}\\ \left(ppm\right) \end{array}$	$\begin{array}{l} \varepsilon_{gx}^{b}\\ \left({}^{\circ }/h\right) \end{array}$	$\begin{array}{l} \varepsilon_{gy}^{b}\\ \left({}^{\circ }/h\right) \end{array}$	$\begin{array}{l} \varepsilon_{gz}^{b}\\ \left({}^{\circ }/h\right) \end{array}$	$\begin{array}{l} \alpha_{gy}^{b}\\ {(}^{\prime \prime }) \end{array}$	$\begin{array}{l} \delta_{gx}^{b}\\ {(}^{\prime \prime }) \end{array}$	$\begin{array}{l} \delta_{gx}^{b}\\ {(}^{\prime \prime }) \end{array}$	$\begin{array}{l} \beta_{gx}^{b}\\ {(}^{\prime \prime }) \end{array}$	$\begin{array}{l} \beta_{gy}^{b}\\ {(}^{\prime \prime }) \end{array}$
1	9.2	7.1	3.4	0.017	0.011	0.021	5.1	3.2	5.6	−3.6	−6.5
2	9.2	6.5	3.2	0.016	0.009	0.019	4.8	3.1	5.3	−4.1	−6.4
3	9.3	7.2	3.7	0.018	0.012	0.021	5.5	4.0	5.2	−3.3	−6.3
Mean	9.233	6.933	3.5	0.017	0.011	0.020	5.1	3.4	5.4	−3.7	−6.3

**Table 5:** Experiment results in rate-biased mode.

	$ARW_{gx}^{b}{(}^{\circ }/\sqrt{h})$		$ARW_{gy}^{b}{(}^{\circ }/\sqrt{h})$		$ARW_{gz}^{b}{(}^{\circ }/\sqrt{h})$	
Angular Velocity	$30^{\circ }/s$	$60^{\circ }/s$	$30^{\circ }/s$	$60^{\circ }/s$	$30^{\circ }/s$	$60^{\circ }/s$
1	0.00041	0.00042	0.00035	0.00037	0.00042	0.00042
2	0.00042	0.00041	0.00037	0.00036	0.00041	0.00043
3	0.00040	0.00039	0.00036	0.00037	0.00041	0.00042
Mean	0.00041	0.00040	0.00036	0.00036	0.00041	0.00042

**Table 6:** Experiment results in mechanically dithered mode.

	$ARW_{gx}^{b}{(}^{\circ }/\sqrt{h})$		$ARW_{gy}^{b}{(}^{\circ }/\sqrt{h})$		$ARW_{gz}^{b}{(}^{\circ }/\sqrt{h})$	
Angular Velocity	$30^{\circ }/s$	$60^{\circ }/s$	$30^{\circ }/s$	$60^{\circ }/s$	$30^{\circ }/s$	$60^{\circ }/s$
1	0.0013	0.0013	0.0012	0.0012	0.0014	0.0011
2	0.0013	0.0013	0.0013	0.0013	0.0012	0.0013
3	0.0012	0.0013	0.0012	0.0013	0.0012	0.0014
Mean	0.0013	0.0013	0.0012	0.0013	0.0013	0.0014

**Table 7:** Results of alignment experiments.

Time (min)	Standard Deviation of Azimuth Angle	
	Rate-Biased Mode (″)	Mechanically Dithered Mode (″)
12	27.9	67.9
16	23.7	64.7
20	21.3	53.9
24	21.2	51.2
28	20.4	47.4
32	18.7	47.8
36	19.1	43.3
40	18.7	42.7
